# Tight adherent feature on optical coherence tomography predict postoperative visual outcome in epiretinal membrane eyes

**DOI:** 10.1186/s12886-022-02569-7

**Published:** 2022-08-18

**Authors:** Tzu-Ting Lai, Li-Li Wu, Yi-Ting Hsieh, Chia-Chen Lee, Yi-Jie Peng

**Affiliations:** 1 Department of Ophthalmology, Taipei Tzu Chi Hospital, The Buddhist Tzu Chi Medical Foundation, Taipei, Taiwan; 2 Department of Ophthalmology, En Chu Kong Hospital, Taipei, Taiwan; 3 Department of Ophthalmology, National Taiwan University Hospital, Taipei, Taiwan; 4 Department of Internal Medicine, National Taiwan University Hospital, Taipei, Taiwan; 5 College of Medicine, Tzu Chi University, Hualien, Taiwan

**Keywords:** ERM, Outer retinal layer, Inner retinal layer, Ectopic inner retinal layer

## Abstract

**Background:**

To identify the predictive parameter among preoperative measurements that best predicts postoperative visual outcome in the epiretinal membrane (ERM).

**Methods:**

Thirty-three consecutive patients with idiopathic unilateral ERM patients between 2015 and 2018 were enrolled. Nineteen healthy normal eyes were selected as an independent age-matched group. Based on preoperative optical coherence tomography (OCT), we further divided the patients with ERM into two groups: type 1, loosely attached ERM, and type 2, tight adherent ERM. We documented the vision and thickness of various retinal layers: nerve fiber layer, ganglion cell layer, inner plexiform layer (GCL + IPL), inner nuclear layer (INL), outer retinal layer (ORL), and retinal pigment epithelium/Bruch complex layer before and after the surgery. The association between postoperative visual acuity and these variables was analyzed using multiple linear regression analysis.

**Results:**

All retinal layers of ERM eyes were thicker than the normal eyes (*P <* 0.05). Among ERMs, we identified 11 eyes with type 1 adhesions and 22 eyes with type 2 adhesions. The preoperative GCL + IPL layers were significantly thicker in type 2 patients than in type 1 patients (93.67 ± 33.03 um vs 167.71 ± 13.77 um; *P =* 0.023). Greater GCL + IPL thickness was correlated with a worse postoperative visual acuity and multiple linear regression analysis showed that GCL + IPL thickness was an independent predictor of postoperative visual acuity (VA) (beta value = 0.689; *P =* 0.012). A greater thickness of GCL + IPL layers of type 2 patients had worse postoperative best-corrected visual acuity (BCVA) (*P =* 0.028). Ectopic inner foveal layers with disappearance of fovea pit were persistently presented in OCT profiles of both groups.

**Conclusion:**

Idiopathic ERM demonstrated significantly thicker inner retinal layers (GCL + IPL and INL). However, the ORL thickness was similar between the normal eyes and ERM eyes. The preoperative GCL + IPL layers were significantly thicker in patients with type 2 ERM than that in patients with type 1 ERM. The increase in GCL + IPL thickness was significantly correlated with worse postoperative visual outcomes.

## Introduction

Epiretinal membrane (ERM), or macular pucker, is an abnormality of fibrotic proliferation at the vitreomacular interface, which causes morphological changes and visual loss. Clinical presentations include metamorphopsia, monocular diplopia, micropia, decreased visual acuity, and loss of central vision.

The pathophysiological evidence suggests that for one-third of the surgically peeled ERMs, the direct interaction between ERM and internal limiting membrane (ILM) causes strong adhesion, which leads to the separation of ERM at the level of the retinal side of the ILM and simultaneous removal of ERM and ILM [1, 2]. In contrast, the residual vitreous cortex induced by incomplete posterior vitreous detachment forms loose adhesion with ILM, which ultimately splits at the vitreal side of the ILM [2, 3]. Although the mechanism of ERM is gradually clear, the precise pathophysiological link to the clinical entity is not completely understood.

Vitreoretinal surgery is a common procedure for the management of ERM. Various classification systems have been proposed for a more adequate diagnosis and better characterization of surgical indications [4, 5]. Improvements in image techniques, including spectral-domain optical coherence tomography (SD-OCT) with better resolution and faster acquisition speed, have facilitated changes in the knowledge of ERM.

Advances in SD-OCT techniques have driven researchers to detect fovea microstructures underneath ERM. Various studies have implicated the role of photoreceptor layer changes associated with ERM [6, 7]. However, mounting published studies have revealed a correlation between the inner retinal layer and visual acuity prognosis [8]. There are still debates on which layers have a more profound impact on visual function and metamorphopsia.

Our study used in-depth SD-OCT images to evaluate the effect of different planes of ERM separation and changes in retinal structure by morphological classification of ERM.

## Materials and methods

### Participants and data collection

In this retrospective study, we collected the medical records of patients with ERM who underwent smooth and uneventful surgery between January 1, 2015, and January 1, 2018, at the Department of Ophthalmology, Taipei Tzu-Chi Hospital. The surgeries were performed by three retinal specialists (YJ Peng, LL Wu, and YT Hsieh). We recruited idiopathic unilateral patients, and the consensus of surgical intervention of the three surgeons was preoperative BCVA worse than 20/40 in the Snellen chart. The procedures were performed using standard 23-gauge transconjunctival three-port pars plana vitrectomy. A core vitrectomy with induction of posterior vitreous detachment, if not already present, was performed. The internal limiting membrane (ILM) was peeled using 0.05% indocyanine green dye. The extent of ERM or ILM peeling was 2–3 optic disc diameters around the fovea. Phacoemulsification with intraocular lens implantation was performed simultaneously if a clinically significant cataract was observed (11 eyes of total 33 ERM groups). During the 1-year postoperative follow-up period, all vitrectomy-related cataract extraction was sequentially performed smoothly (*N =* 22). We excluded data from patients with 1) ERM after previous retinal detachment surgery, 2) coexisting diabetic retinopathy, retinal vein occlusion, or uveitis; 3) previous trauma history; 4) cataract which significantly influenced visual function and 5) high myopic eyes with spherical equivalent ≤ -6.0 D. BCVA was measured and documented using a Snellen chart preoperatively and 1 year after surgery. All measurements were converted into the logarithm of the minimal angle of resolution (LogMAR) values for statistical analysis. At the end of one year follow-up after vitrectomy procedure, the final SD-OCT sections of the macular region were collected for further analysis.

### Image acquisition and data analysis

**Fig. 1:**
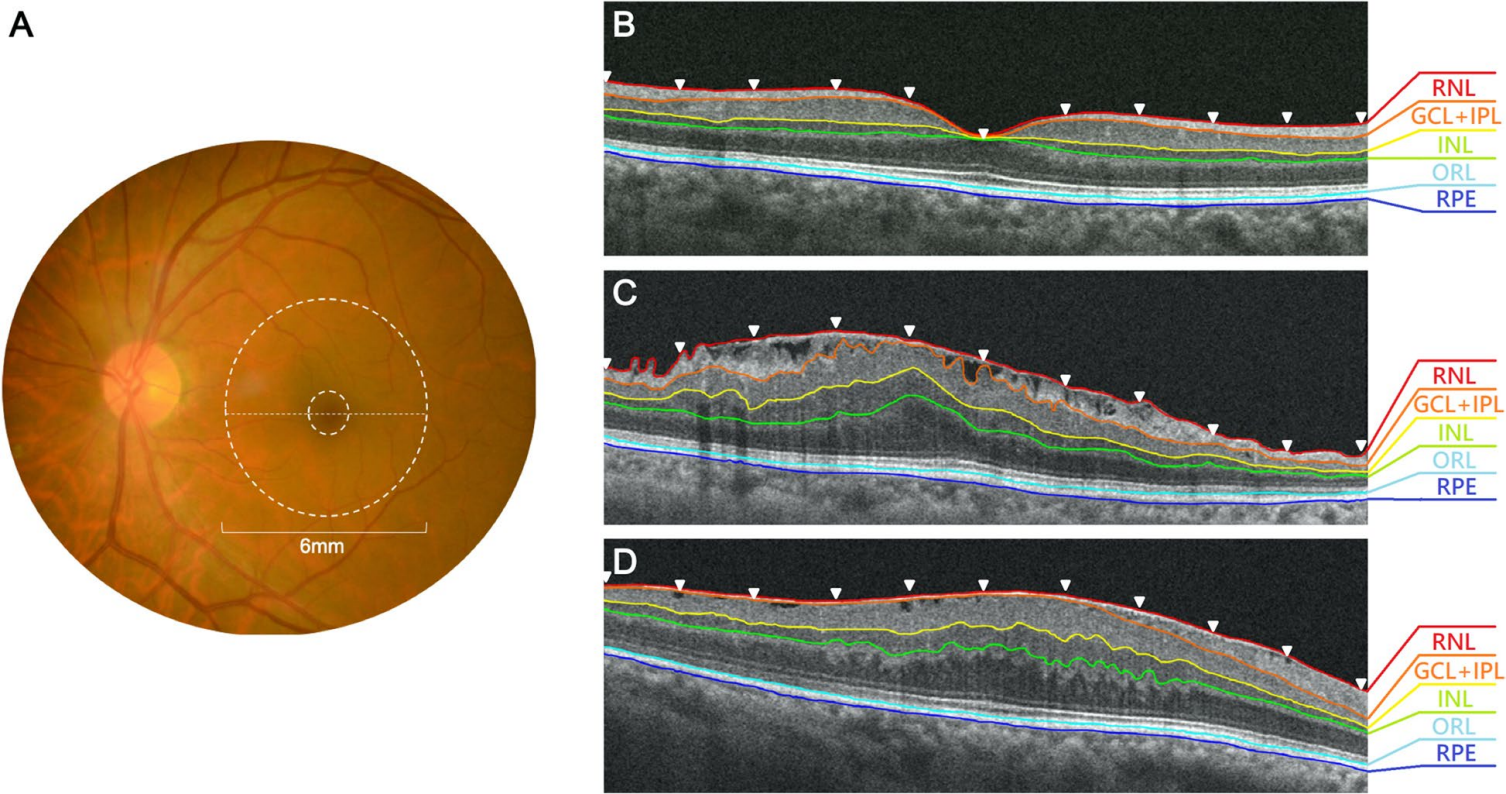
Images of fundus color and SD-OCT results of manual segmentation of macular intraretinal layers. **A** The representative 6-mm circular region in the macula was analyzed as thickness profiles of different intraretinal layers. **B** the Cross- sectional image along the horizontal meridian of a normal eye. **C** Cross-sectional image along the horizontal meridian of Type I loosely attached ERM eye. **D** Cross-sectional image of Type II globally adherent ERM eye

OCT was performed using Cirrus high-definition optical OCT (HD-OCT; Carl Zeiss Meditec, Dublin, CA, USA). The images were generated using a horizontal meridian cross-section with a quality score > 7. The central 6-mm circular region in the macula was selected, and a representative section (horizontal dotted line in Fig. 1) was selected for further retinal layer thickness analysis. Each section was evenly divided into 600 ums wide sections as the 11 white arrowheads shown in Fig. 1. These 11 white arrowheads mark the measuring points, where the (1) nerve fiber layer (NFL), (2) ganglion cell layer and inner plexiform layer (GCL + IPL); (3) inner nuclear layer (INL); (4) outer retinal layer (ORL); and (5) retinal pigment epithelium/ Bruch complex layer were identified, and the interface between these layers was manually marked by two independent observers [9]. OCT images containing retinoschisis or large retinal cysts and significant retinal layer disorganization were excluded. The distance between the interfaces was measured automatically by intrinsic Zeiss CIRRUS software algorithms and documented as the thickness of the five retinal layers. Values were averaged for the statistical analysis.

We divided the patients with ERM into two groups based on the OCT image characteristics: type 1, the membrane is loosely attached with several point adhesions, and optical lucent spaces can be easily seen between the ERM and the inner surface of the retina. Type 2: The membrane is tightly adherent to the surface of the retina, and only a few small optical lucent areas can be detected between the ERM and the inner surface of the retina. The length of ERM-retinal adhesion length was measured using an electronic caliper in the HD-OCT instrument. Based on the Kim’s study [3], the ratio was calculated by dividing the length without plication by the length of the ERM. The extent of ERM-retinal adhesion was defined as a firm adhesion of > 70% and loose adhesion of < 70%. We chose the inner border of the retina at the outer margin of the optical lucent spaces as the inner border of the NFL in Type 1 patients, and the outer margin of the ERM as the inner border of the NFL in Type 2 patients.

### Statistical analysis

Data were expressed as mean ± standard deviation and analyzed using SPSS software (version 22.0; SPSS, Inc., Chicago, IL, USA). Statistical significance was set at *P <* 0.05. The Kolmogorov–Smirnov test was used to verify the distribution normality for continuous variables. The comparison between type 1 and type 2 preoperative and postoperative parameters was assessed using independent t-tests and paired t-tests. Univariate linear regression was performed to identify the association between BCVA and independent variables, including intraretinal layer thickness and outer retinal layer disruption.

## Results

### Demographics

A total of 33 eyes were recruited from the ERM group and 19 phakic eyes in the age-matched control group. There were no significant differences in age and sex between the control and ERM groups (Table 1). In total 33 eyes of ERM group, 11 eyes (33.3%) had received concomitant cataract surgery and remaining 22 eyes (66.7%) underwent cataract extraction during the 1-year postoperative follow-up period. Patients with cataract which significantly influenced visual function was excluded. In the ERM group, 11 eyes presented with loosely attached ERMs and were categorized as type 1. The other 22 eyes presented with tight adherent ERMs and were categorized as type 2.

**Table 1:** Subjective characteristics of Normal and ERM eyes

	**Normal eyes**	**ERM eyes**	***P* value**
**NO of eye**	19	33	
**Age**	65.20 ± 8.62	64 ± 8.96	0.675
**Sex (F/M)**	10/9	25/8	0.216
**Phakic Lens status (%)**	64.7	100	< 0.05
**IS/OS disruption (%)**	0	18.8	
**ELM disruption (%)**	0	9.4	
**Cost disruption (%)**	0	25	
**Cyst (%)**	0	31.3	
**Pre-OP BCVA**	0.02 ± 0.04	0.77 ± 0.05	0.02
**Post-OP BCVA**		0.41 ± 0.04	
**Pre-OP GCL + IPL**		149.77 ± 12.79	
**Pre-OP INL**		103.48 ± 11.31	
**Pre-OP ORL**	107.88 ± 3.5	157.58 ± 13.77	0.01

*Abbreviation*: *IS/OS* Inner/outer segment, *ELM* External limiting membrane, *COST* Cone outer segment tips, *BCVA* Best corrected visual acuity, *OP* Operation, *GCL* + *IPL* Ganglion cell layer and inner plexiform layer, *INL* Inner nuclear layer, *ORL* Outer retinal layer

The thickness of Pre-OP GCL + IPL, INL, ORL were measured at fovea

The mean logarithm of the minimum angle of resolution post-operational BCVA was 0.33 ± 0.05 in type 1 ERM eyes and 0.51 ± 0.06 in type 2 ERM eyes. There was statistically significance of visual acuity between both ERM groups (Table 2, *P =* 0.028).

**Table 2:** Optical coherence tomography parameters in eyes that underwent epiretinal membrane surgery between two ERM groups

Anatomic characteristics of ERM eyes					
	Loose attached type I		Tight adhesion type II		*P* value^b^
		*P* value^a^		*P* value^a^	
**No of eye**	11		22		
**Pre-GCL + IPL (um)**	93.67 ± 33.03		167.71 ± 13.77		0.023
** Pre-OP**	93.10 ± 23.06	0.09	169.11 ± 11.33	< 0.001	
** Post-OP**	51.4 ± 10.96		73.90 ± 9.40		
** Df OP**	41.7 ± 22.58		114.48 ± 17.24		0.02
**INL (um)**	69.83 ± 25.81		129.64 ± 17.84		0.086
** Pre-OP**	69.7 ± 17.17	0.382	119.62 ± 14.51	0.008	
** Post-OP**	60.6 ± 13.19		67.53 ± 9.71		
** Df OP**	15.8 ± 12.5		61.43 ± 16.62		0.087
**ORL (um)**	145.17 ± 51.9		155.14 ± 17.04		0.815
** Pre-OP**	151.3 ± 30.6	0.651	162.30 ± 15.42	0.468	
** Post-OP**	137.3 ± 10.42		148.14 ± 10.90		
** Df OP**	14 ± 29.92		44.05 ± 20.69		0.416
**CRT (um)**	451.7 ± 19.49		536.19 ± 15.78		0.01
** Pre-OP**	512 ± 17.14	< 0.001	540.67 ± 20.08	< 0.001	
** Post-OP**	379 ± 15.29		388.67 ± 14.82		
** Df OP**	88.45 ± 14.64		148.77 ± 18.41		0.04
**Visual Characteristics of ERM eyes**					
					*P* value
**Pre-BCVA (LogMAR)**	0.67 ± 0.10		0.86 ± 0.08		0.182
**Post-BCVA(LogMAR)**	0.33 ± 0.05		0.51 ± 0.06		0.028

^a^Paired t-test between pre-operational and post-operational thickness

^b^Independent t-tests between type I and type II ERM groups

*Abbreviation*: *BCVA* Best corrected visual acuity, *GCL* + *IPL* Ganglion cell layer and inner plexiform layer, *INL* Inner nuclear layer, *ORL* Outer retinal layer, *CRT* Central fovea thickness, *LogMAR* Logarithm of the Minimum Angle of Resolution

### Intraretinal structure analysis

**Fig. 2:**
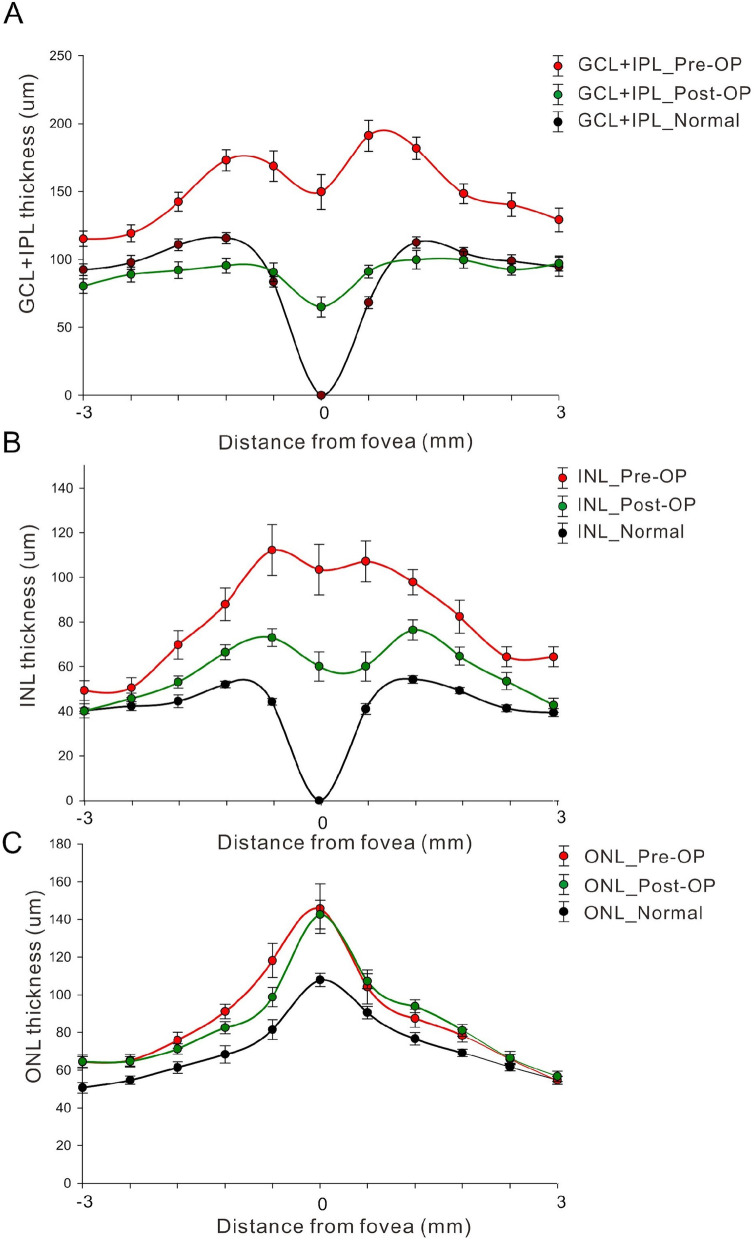
Segmentation profiles of intraretinal layers in normal eyes and ERM eyes. **A**, **B** The pre-operational inner retinal thickness (GCL + IPL and INL respectively) was much thicker in the epiretinal membrane group compared with normal eyes and post-operational thickness. The ganglion cell layer and inner nuclear layer showed abnormal displacement of spatial organization at fovea. **C** The ORL thickness was no significant difference between these two groups

**Fig. 3:**
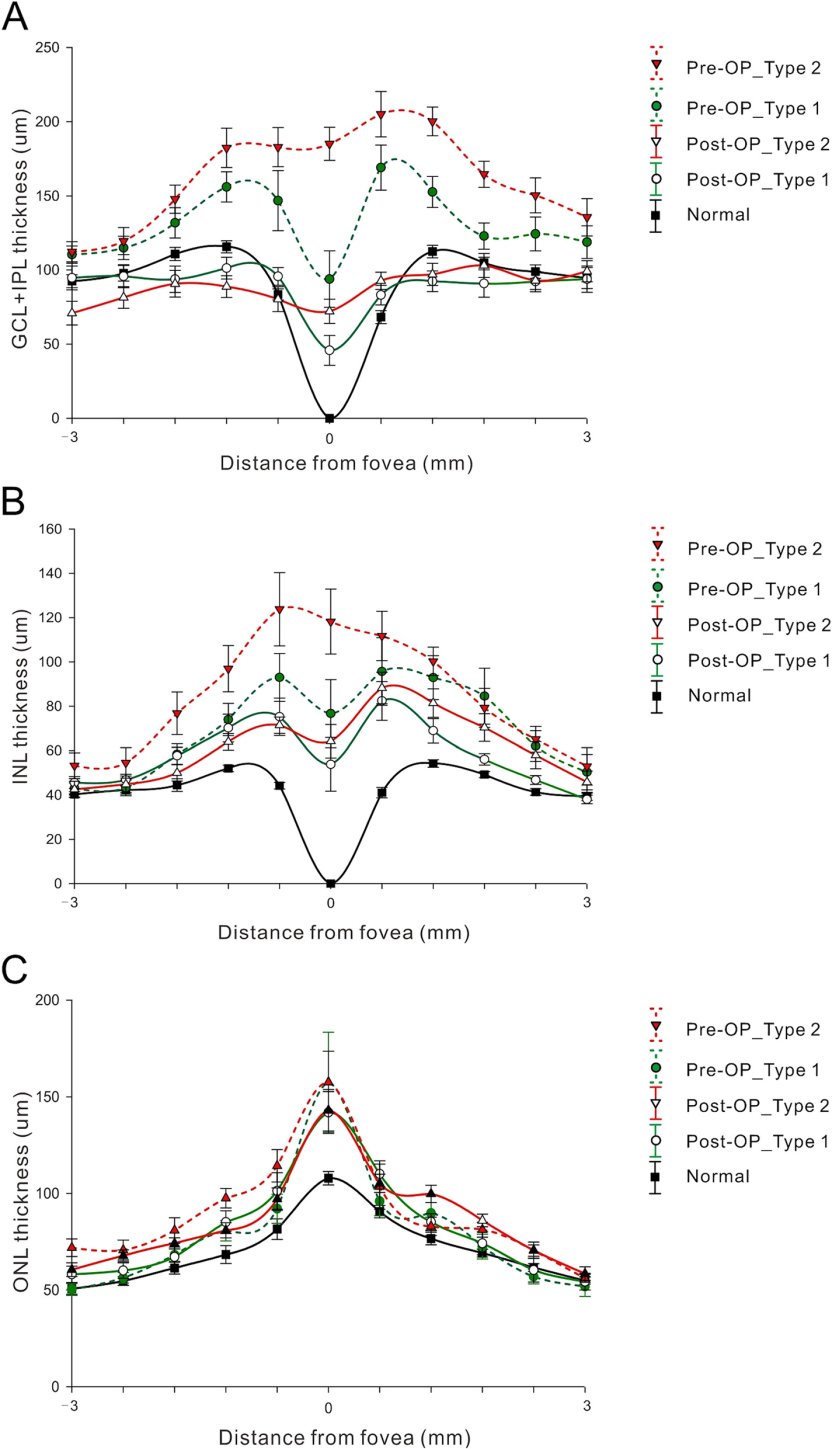
Thickness profiles of different intra-retinal layers measured pre-operatively and post-operatively between different groups. The pre-operational GCL + IPL/INL thickness in ERM type II globally adherent group was larger than those of ERM type I group and normal eyes. The post-operational thickness of GCL + IPL/INL was tended to be normal. However, the outer nuclear layer had no difference to normal group

The selected retinal layer thicknesses (GCL + IPL, INL, and ORL) of the three groups are shown in Fig. 2. The abnormal presence of GCL + IPL and INL at the central most measuring point, presumably to be the location of the fovea, can be found in both ERM groups (Fig. 2A and B). The average GCL + IPL thickness was significantly thicker in eyes with type 2 ERM (Fig. 3A, *P* = 0.023) than in type 1 ERM eyes. We also identified a difference in INL thickness between the two groups (Fig. 3B). (*P =* 0.086).

We further analyzed the postoperative changes in these layers to determine whether the membrane peeling procedure could restore the foveal contour. The GCL + IPL and INL thickness in both ERM groups significantly decreased, especially in type 2 ERM eyes; however, persisted at the central fovea after the surgery (Fig. 3A, B and Table 2, *P <* 0.001 and *P =* 0.008 for GCL + IPL and INL, respectively). We defined the continuous retinal layers extending from the GCL + IPL to the INL on OCT across the fovea as the fovea-occupying ectopic inner retinal layer. In normal controls, these layers did not exist at the fovea (thickness = 0). There was no difference in ORL thickness after surgery between the ERM groups. (Fig. 3C and Table 2, *P =* 0.651 and *P =* 0.468 for type 1 and 2, respectively).

### Association between inner retinal layers thickness and BCVA

Correlation analysis suggested that increased GCL + IPL thickness was associated with reduced postoperative BCVA (rho = 0.386, *P =* 0.032) (Table 3). No associations were identified among any other preoperative parameters, including pre-operational INL, ORL, IS/OS, cost, and cyst. Multiple stepwise regression analysis confirmed that GCL + IPL was an independent prognostic predictor of postoperative BCVA (Table 4, *P =* 0.012).

**Table 3:** Correlation of intraretinal layers (um) and different biomarkers with Post-operational BCVA (LogMAR) (*N =* 33)

Dependent	Independent	rho value	*P* value
Post-OP BCVA	Pre-OP GCL + IPL	0.386	0.032
	Pre-OP INL	0.188	0.312
	Pre-OP ORL	-0.161	0.388
	Pre-OP CRT	0.155	0.389
	ELM	0.235	0.188
	IS/OS	-0.221	0.217
	Cost	-0.202	0.258
	Cyst	-0.098	0.588

*Abbreviation*: *IS/OS* Inner/outer segment, *ELM* External limiting membrane, *COST* Cone outer segment tips, *BCVA* Best corrected visual acuity, *OP* Operation, *GCL* + *IPL* Ganglion cell layer and inner plexiform layer, *INL* Inner nuclear layer, *ORL* Outer retinal layer, *LogMAR* Logarithm of the Minimum Angle of Resolution

**Table 4:** Results of multiple linear regression analysis for independent factors contributing to post-operational BCVA (LogMAR) (*N =* 33)

Variables		Standardized	
**Dependent**	**Independent**	**ℬ value**	***P* value**
Post-OP BCVA	Pre-OP GCL + IPL	0.689	0.012
	Pre-OP INL	-0.05	0.901
	Pre-OP ORL	-0.087	0.830

*Abbreviation*: *BCVA* Best corrected visual acuity, *OP* Operation, *GCL* + *IPL* Ganglion cell layer and inner plexiform layer, *INL* Inner nuclear layer, *ORL* Outer retinal layer, *LogMAR* Logarithm of the Minimum Angle of Resolution

## Discussion

ERM-induced tractional force can involve a broad spectrum of macular complications, ranging from thickened retinal layers, macular schisis to the formation of lamellar holes, or full-thickness macular holes. In our study, we identified an abnormal centripetal displacement of inner foveal layers by ERM traction. There is significant thickening in the inner retinal layers and a dramatic thickness reduction of the inner retinal layers after surgery. These results revealed that persistent mechanical traction in ERM affected both the inner retinal thickness and morphological retinal structure [10]. Furthermore, a greater thickness of the GCL + IPL played an important role in predicting worse postoperative BCVA performance.

The exact mechanism by which the epiretinal membrane exerts traction at the vitreoretinal surface remains unknown. There is pathological evidence that despite evidence of a Weiss ring, remnants of the vitreous cortex can still be found on the retinal surface, indicating the presence of vitreoschisis. Gandofer et al. further proposed two different separation planes can cause different types of ERM traction [2]; One type of traction is that in the presence of vitreoschisis, ERM was proliferated on top of the residual vitreous cortex, and ILM was left uninvolved underneath the vitreous cortex during membrane peeling. This type of traction demonstrated with loose vitreoretinal attachment and gaps between the ILM and ERM might be identified on SD-OCT. The other traction occurs when the ERM directly adheres to the ILM. The second type of adhesion should exert stronger traction on the retina and increase the macular structure. Therefore, based on the pathological observations, we applied topographic features of the vitreoretinal surface to classify the traction pattern and correlate it with disease severity.

Tangential traction of ERM may lead to retinal surface folding, thickening of inner retinal layers and centripetal displacement of inner retina. The traction of ERM directly forced movement of the underlying inner retinal layers towards the foveal center resulting in disappearance of fovea pit and elevation of inner retinal layer of foveal walls [11]. The importance of traction from ERM for thickening of the inner retinal layers is manifested by the data shown in Fig. 2 in which only GCL + IPL and INL were thickened but not ORL. As for the inner retinal layer, the more proximal layers near ERM, was the primary affected site of ERM-associated mechanical force. The strength of mechanical stress is in proportion to thickening of retinal layers. Our study demonstrated significantly thicker GCL + IPL thickness and INL thickness in patients with type 2 ERM than in type 1 patients. This is compatible with our hypothesis that the tight adherent epiretinal membrane exerts more anteroposterior and tangential force directly to the proximal inner retina. This imbalance in thickness contributes to the cause of visual impairment.

There is controversary regarding prognostic factors of visual outcome after ERM surgery. The integrity of photoreceptors and ellipsoid zone disruption were identified to be correlated with visual acuity [6]. More recent studies revealed the role of inner retinal layers in vision loss. GCL + IPL layer thickening was significantly related to poor visual acuity in many reports [10, 12]. Our study showed that postoperative visual acuity was inversely correlated with the thickness of the GCL + IPL layer (Table 4).

The decreased acuity of ERM eyes was also associated with the ectopic inner foveal layers and disappearance of fovea pit which changes the light transmission and spatial resolution through the fovea [13]. During development, centrifugal migration of inner retinal neurons is necessary to differentiate the fovea from the surrounding retina and reduce the light scattering to ensure sharp visual acuity [14]. The ERM-mediated displacement of inner retinal layers is a pathologically inverse process compared to developmental eyes and mal-adapted contour revision under ERM traction. Our results showed ERM-induced centripetal displacement of inner retinal layers including GCL + IPL and INL across the central fovea and this ectopic inner retinal layer does not restore even after ERM peeling. Our study also suggested clinical implication that the presence of thicker ectopic inner foveal layer was accompanied with worse vision acuity and limited postoperative visual recovery. In addition, the higher index of suspicion for surgical-induced retinal injury should exist that tight adhesion ERM bridging retinal surface exerts stronger force over underlying retina. Together with these results, the visual acuity was dependent on both thickness of the inner retina and the integrity of photoreceptors.

The inner retinal thickness decreased after vitrectomy with ERM peeling, as in our case, the presence of ectopic inner retinal layer persisted with the decreased foveal thickness over postoperative period. There were still unknown causes for the failure of complete recovery of normal fovea shape after ERM peeling. One reason was upregulation of glial intermediate filaments and GFAP of muller cells increases the stiffness of the tissue and impairs the competence of movement [15]. Another possible explanation was muller cells were pulled by the ERM traction centripetally and this impaired the centrifugal tissue movement for normal foveal contour.

There are several limitations of our study. Firstly, this study was small sample size, especially after excluding cataract interference. Secondly, ERM generates uneven traction over different meridians, but our OCT images were measured only along scan’s x axis. The lack of information on retinal thickness at different meridians could be a limitation of our study. Finally, severe ERM traction causes foveaschisis and significant retinal layer disorganization. Segmentation failure for each retinal layer often occurred and we did exclude cases from artifact analysis. This could lead to selection bias and loss some pathological steps in the developmental ERM. Therefore, our findings that preoperative thicker GCL + IPL layers in type 2 ERM was a prognostic factor for the degree of VA damage are only applicable to patients with relatively intact retinal layers.

The inner retina, particularly the GCL + IPL layer, has a larger thickness in idiopathic ERM and can be regarded as a prognostic factor for postoperative visual acuity. The fovea-occupying ectopic inner retinal layer was also worth noting in the OCT-based thickness profile. These results suggest the importance of inner retinal layers in the visual outcome of ERM.

Our study is one of the first to apply the morphological classification of SD-OCT images to ERM patients and found that type 2 tight adherent ERM patients truly exhibit thicker GCL + IPL thickness than type 1 loosely attached ERM patients. Obtaining the GCL + IPL thickness data can be time-consuming and thickening or abnormal displacement of the layer can be ambiguous to the parafovea normal retinal structure in real-world clinical settings. The clinical significance of this study is to help clinicians to speculate which group of patients may yield better anatomical and visual outcomes at the first glance of the OCT image.

## Data Availability

All data generated or analyzed during this study are included in this published article.
